# Ultrasonic Particle Manipulation in Glass Capillaries: A Concise Review

**DOI:** 10.3390/mi12080876

**Published:** 2021-07-26

**Authors:** Guotian Liu, Junjun Lei, Feng Cheng, Kemin Li, Xuanrong Ji, Zhigang Huang, Zhongning Guo

**Affiliations:** 1 State Key Laboratory of Precision Electronic Manufacturing Technology and Equipment, Guangdong University of Technology, Guangzhou 510006, China; lgt15814518508@163.com (G.L.); cff951151311@163.com (F.C.); lkm1300454596@163.com (K.L.); xr.ji@gdut.edu.cn (X.J.); huangzg@gdut.edu.cn (Z.H.); znguo@gdut.edu.cn (Z.G.); 2 Guangzhou Key Laboratory of Non-Traditional Manufacturing Technology and Equipment, Guangdong University of Technology, Guangzhou 510006, China

**Keywords:** ultrasonic particle manipulation, acoustic tweezers, acoustic radiation force, acoustic streaming, glass capillary, miniaturized ultrasonic devices

## Abstract

Ultrasonic particle manipulation (UPM), a non-contact and label-free method that uses ultrasonic waves to manipulate micro- or nano-scale particles, has recently gained significant attention in the microfluidics community. Moreover, glass is optically transparent and has dimensional stability, distinct acoustic impedance to water and a high acoustic quality factor, making it an excellent material for constructing chambers for ultrasonic resonators. Over the past several decades, glass capillaries are increasingly designed for a variety of UPMs, e.g., patterning, focusing, trapping and transporting of micron or submicron particles. Herein, we review established and emerging glass capillary-transducer devices, describing their underlying mechanisms of operation, with special emphasis on the application of glass capillaries with fluid channels of various cross-sections (i.e., rectangular, square and circular) on UPM. We believe that this review will provide a superior guidance for the design of glass capillary-based UPM devices for acoustic tweezers-based research.

## 1. Introduction

Controlled manipulation of microparticles such as bacteria and cells is important for both fundamental research and applications in the fields of engineering, physics, biomedicine and chemistry. To date, numerous microfluidics technologies, such as electrical [1], magnetic [2], optical [3], hydrodynamic [4] and acoustic [5] methods, have been proposed and developed for the manipulation of microparticles. Among the many techniques, acoustic particle manipulation enabled by acoustic radiation force [6] or acoustic streaming [7] has been proved to be a promising method as it requires no pretreatment of the particles (i.e., is a label-free manipulation method), can manipulate particles without contact and regardless of their electric, optical or magnetic properties, and has little effect on the viability of cells [8,9,10].

The acoustic particle manipulation device is commonly referred to as acoustic tweezers [11]. One of the main components of acoustic tweezers is the acoustic transducer, which converts electrical energy into mechanical vibrations and generates sound waves in fluid channels that are used to manipulate particles [12]. The most frequently used transducers are piezoelectric ceramic transducers (PZTs) and interdigital transducers (IDTs), which are mainly used to generate bulk acoustic waves (BAWs) and surface acoustic waves (SAWs), respectively. In practice, the transducers of most acoustic tweezers are generally actuated by ultrasonic frequencies (i.e., ultrasonic particle manipulation (UPM) [13]), more usually at mega Hertz. According to different manipulation mechanisms, acoustic tweezers can generally be divided into three main categories [14], i.e., standing-wave tweezers, travelling-wave tweezers and acoustic-streaming tweezers.

When the standing wave technology (which is the most frequently used category) is applied, the acoustic particle manipulation devices are also referred to as acoustic resonators [15]. A fundamental requirement to accomplish various types of particle manipulation (e.g., patterning, trapping and separation) is to design well-defined acoustic resonators. This is closely related to another important component of acoustic tweezers, i.e., the microfluidics system, which mainly includes the fluid channel and the surrounding materials. The surrounding materials of fluid channels are crucial to the performance of acoustic resonators as they determine the propagation and attenuation of acoustic waves. The fluidic channels of common acoustic resonators are made out of a variety of materials, such as glass, silicon, metal, polymer (mostly PDMS) and paper. Polymer materials do not have good acoustic reflection performance and are usually used in SAW microfluidics [16]. Glass, silicon and metal, however, have low acoustic attenuation and high difference in acoustic impedance compared with fluid, which are beneficial to the effective propagation of acoustic waves and the formation of strong acoustic resonances, and are usually used in BAW resonators. Specifically, glass, due to its excellent optical transparency, uniquely enables observation of microparticle acoustophoresis in the fluid channel from any direction, making it an excellent material for constructing chambers for ultrasonic resonators. Moreover, glass capillaries, occasions that make it easy to generate whole resonances and have no requirement on additional bonding of a glass layer (i.e., sealing of the channel in width and height directions), have been widely used in UPM in the past decades.

**Figure 1:**
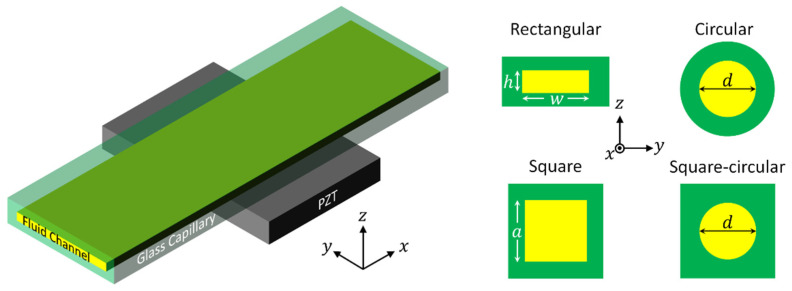
Schematic presentation of a typical glass capillary-based ultrasonic particle manipulation device, which mainly consists of an ultrasonic transducer (here PZT) and a glass capillary. The common cross-sections of glass capillaries designed for ultrasonic particle manipulation are schematically shown on the right side, where square-circular indicates glass capillaries whose outer and inner cross-sections are square and circular, respectively.

In this work, we present a concise review on the recent advancement of glass capillary-based UPM devices. Unlike many previously published reviews on acoustic tweezers based on the classification of manipulation principles, e.g., [17,18,19,20,21,22], this review emphasizes on UPM in glass capillaries with fluid channels of various cross-section structures, mainly rectangular, square and circular, which are shown schematically in Figure 1. A brief description of different applications that have been achieved by each type of glass capillary is introduced.

## 2. Theory of Ultrasonic Particle Manipulation (UPM)

In an UPM device, two main acoustic forces, i.e., the acoustic radiation force and the acoustic streaming-induced drag force, act on the particles suspended in the fluid. To calculate these two forces, spatial distributions of the acoustic field and the acoustic streaming field have to be clear. Here, we briefly introduce the perturbation theory, the most common method that has been used to analyze the acoustic and streaming fields in UPM devices [23,24]. In the following, bold and normal-emphasis fonts are used to represent vectors and scalar quantities, respectively.

For a homogeneous isotropic fluid, the continuity and momentum equations for the fluid motion are

(1) $$\frac{\partial \rho }{\partial t}+\nabla \cdot \left(\rho u\right)=0,$$

(2) $$\rho \left(\frac{\partial u}{\partial t}+u\cdot \nabla u\right)=-\nabla p+\mu \nabla^{2}u+\left(\mu_{b}+\frac{1}{3}\mu \right)\nabla \nabla \cdot u,$$

where ρ is the fluid density, t is time, u is the fluid velocity, p is the pressure, and μ and $\mu_{b}$ are the dynamic and bulk viscosity coefficients of the fluid, respectively. By using the perturbation theory, we can write the fluid density ρ, pressure p, and velocity u in the form

(3) $$\rho =\rho_{0}+\rho_{1}+\rho_{2}+\cdots ,$$

(4) $$p=p_{0}+p_{1}+p_{2}+\cdots ,$$

(5) $$u=u_{1}+u_{2}+\cdots ,$$

where the subscripts 0, 1 and 2 represent the static (i.e., absence of ultrasonic excitation), first-order and second-order quantities, respectively.

Substituting Equations (3)–(5) into Equations (1) and (2) and taking the first-order into account, Equations (1) and (2) become

(6) $$\frac{\partial \rho_{1}}{\partial t}+\rho_{0}\nabla \cdot u_{1}=0,$$

(7) $$\rho_{0}\frac{\partial u_{1}}{\partial t}=-\nabla p_{1}+\mu \nabla^{2}u_{1}+\left(\mu_{b}+\frac{1}{3}\mu \right)\nabla \nabla \cdot u_{1}.$$

To obtain equations for acoustic streaming from Equations (1) and (2), we keep terms up to second order and take the time average of the continuity and momentum equations. Equations (1) and (2) are then turned into

(8) $$\nabla \cdot \bar{\rho_{1}u_{1}}+\rho_{0}\nabla \cdot \bar{u_{2}}=0,$$

(9) $$-\rho_{0}\bar{u_{1}\nabla \cdot u_{1}+u_{1}\cdot \nabla u_{1}}=-\nabla \bar{p_{2}}+\mu \nabla^{2}\bar{u_{2}}+\left(\mu_{b}+\frac{1}{3}\mu \right)\nabla \nabla \cdot \bar{u_{2}},$$

where the upper bar $\bar{\cdot }$ indicates the time-averaged value of the quantity below.

In most UPM devices where usually only the outer streaming fields are of interest, Equations (8) and (9) can be further simplified to

(10) $$\nabla \cdot \bar{u_{2}}=0,$$

(11) $$-\nabla \bar{p_{2}}+\mu \nabla^{2}\bar{u_{2}}=0.$$

The equations above describe the fundamental theory of acoustofluidics. In practice, these equations are usually solved using numerical simulations, which cannot only be used to predict the acoustofluidic fields and the performance of microparticles to assist device design but can also be applied to validate or to explain the complex phenomena observed in UPM devices. In general, the whole procedure to model the acoustophoretic motion of microparticles can be divided into the following three main steps:

(1) Simulation of the acoustic fields.

In most UPM devices, the acoustic pressure $p_{1}$ is within the linear acoustic regime such that it can be solved from the linear Helmholtz equation [25], i.e.,

(12) $$\nabla^{2}p_{1}+\frac{\omega^{2}}{c_{0}^{2}}p_{1}=0,$$

where ω is the angular frequency and $c_{0}$ is the speed of sound in the fluid. Then, the acoustic velocity field can be determined from the linearized Euler’s equation [25], following

(13) $$u_{1}=-\frac{i}{\rho_{0}\omega }\nabla p_{1},$$

where $\rho_{0}$ is the static fluid density and $i=\sqrt{-1}$ is the imaginary unit. This equation is applicable to conditions where the magnitude of the acoustic particle velocity is small compared with the sound speed in the fluid, i.e., $\left|u_{1}\right|\ll c_{0}$ (which can also be obtained from $\rho_{1}\ll \rho_{0}$).

(2) Simulation of acoustic streaming fields.

In most UPM devices, the acoustic streaming fields are dominated by the boundary-driven acoustic streaming. As shown above, the solution of acoustic streaming by solving Equations (8) and (9) is usually called the Reynolds stress method, which solves both inner and outer acoustic streaming fields in a fluid channel. Equations (10) and (11) can be applied to effectively solve the outer acoustic streaming fields, if a limiting velocity (equation of the linear acoustic velocity field) [26] is applied as a slip-velocity boundary condition to the fluid–solid interfaces where the viscous boundary layer is ignored. The method of solving the outer acoustic streaming fields by using the limiting velocities is known as the limiting velocity method. A comparison of these two methods for the modeling of boundary-driven acoustic streaming fields in fluid channels of rectangular cross-section was recently studied by Lei et al. [27]. 

Generally speaking, the Reynolds stress method is more accurate as it takes into account the thin boundary layer and solves acoustic streaming from its genesis, the Reynolds stress force, i.e., the left-hand-side of Equation (9), while the limiting velocity method is more computationally efficient and is suitable for three-dimensional (3D) simulations. In the past decade, with the assistance of the limiting velocity method, a number of acoustic streaming patterns, including the classical Rayleigh-type streaming [28] and new (i.e., those that cannot be explained by classical Rayleigh streaming theory [29]) streaming such as Modal Rayleigh-like streaming [30] and transducer-plane streaming (e.g., four-quadrant [31,32,33] and eight-octant [34] patterns, which are usually seen in planar resonant devices [35]) in glass capillaries, have been modeled and elucidated through 3D simulations.

(3) Simulation of microparticle acoustophoresis.

Having obtained acoustic and streaming fields, the acoustic radiation force and acoustic streaming-induced drag force can then be determined from the Gorkov equation [36] and the Stokes’ law [37], respectively,

(14) $$F_{ac}=\nabla \left\{\frac{4\pi r^{3}}{3}\left[\frac{3\left(\rho_{p}-\rho_{0}\right)}{2\rho_{p}+\rho_{0}}\bar{E_{kin}}-\left(1-\frac{\rho_{0}c_{0}^{2}}{\rho_{p}c_{p}^{2}}\right)\bar{E_{pot}}\right]\right\},$$

(15) $$F_{d}=6\mu \pi r\left(\bar{u_{2}}-v\right),$$

where r is particle radius, $\bar{E_{kin}}=\rho_{0}{\left|u_{1}\right|}^{2}/4$ and $\bar{E_{pot}}={\left|p_{1}\right|}^{2}/\left(4\rho_{0}c_{0}^{2}\right)$ are the time-averaged kinematic and potential energy density, $\rho_{p}$ and ρ_0_ are the density of the particle and fluid, $c_{p}$ and $c_{0}$ are the sound speed in particle and fluid, and ***v*** is the particle velocity. Ignoring the Gravity and the Buoyancy forces, the velocity of microparticles suspended in the fluid is governed by

(16) $$\frac{d}{dt}\left(m_{p}v\right)=F_{ac}+F_{d},$$

where $m_{p}$ is the particle mass. Combining Equations (14)–(16), together with proper initial conditions, the velocities and trajectories of particles at any time can be modeled.

It can be seen from Equations (14) and (15) that the acoustic radiation and streaming-induced drag forces are proportional to $r^{3}$ and r, respectively, indicating that, for a certain acoustic field, there exists a marginal particle radius $r_{m}$ that can make a balance of the magnitudes of these two forces, i.e., $\left|F_{ac}\right|=\left|F_{d}\right|$. Moreover, the acoustic radiation force dominates the acoustophoretic motion of $r\gg r_{m}$ particles while for particles of $r\ll r_{m}$ their trajectories are determined by the acoustic streaming-induced drag force. For example, in a one-dimensional (1D) standing wave field with a driving frequency f=2 MHz, the marginal particle radius $r_{m}\approx 1.6$ µm. For most standing-wave tweezers, acoustic radiation force is the main engine for particle manipulation (e.g., ultrasonic patterning, alignment and separation of microparticles) while acoustic streaming is usually unwanted and generally considered as a ‘disturbance’ to particle manipulation. However, acoustic streaming in some certain circumstances can also play an active role in the trapping and concentration of micron or submicron particles [38,39].

## 3. Ultrasonic Particle Manipulation (UPM) in Glass Capillaries

In the following, we describe recent advancement in glass capillary-based UPM devices, which are typically standing-wave tweezers. In general, most particles and cells of interest are denser and less compressible than typical suspending fluid, so the acoustic radiation force tends to move them to the acoustic pressure nodes (i.e., minima), and the acoustic velocity antinodes (i.e., maxima), as also described in Equation (14). These devices are categorized according to the cross-section of fluid channels. For micro- or nano-scale particle manipulation, the characteristic size (e.g., *w*, *h*, and *d* shown in Figure 1) of the fluid channel is generally of the order of 1 mm or at the submillimeter scale.

It is worth noting that the glass capillaries mentioned here refer to glass chips containing whole fluid channels that require no additional bonding of different components (i.e., sealing of the channel in width and height directions), as shown in Figure 1. Therefore, UPM in microfluidic channels, which are made out of glass and bonded to another glass layer, is not within the scope of this review.

### 3.1. Rectangular Cross-Section Channel Glass Capillary

In rectangular cross-section glass capillaries, a variety of UPMs, such as particle concentration, alignment, patterning, trapping and transportation, have been demonstrated in literature. In a rectangular cross-section fluid channel, the resonant frequency is closely related to the channel dimensions. For example, for a standing wave established in the y-direction of the fluid channel, $f_{y}=c_{0}/\lambda_{y}=c_{0}n_{y}/\left(2w\right)$, where *f*, *λ* and *n* represent, respectively, the resonant frequency, acoustic wavelength and the number of acoustic pressure nodes with subscript y denoting the direction. Similarly, we have $f_{z}=c_{0}/\lambda_{z}=c_{0}n_{z}/\left(2h\right)$ for a standing wave in the *z*-direction. To establish two-dimensional (2D) acoustic resonances in cross-section of the fluid channel, the resonant frequency satisfies

(17) $$f_{y,z}=\frac{c_{0}}{2}\sqrt{{\left(\frac{n_{y}}{w}\right)}^{2}+{\left(\frac{n_{z}}{h}\right)}^{2}}.$$

This equation also covers the 1D standing wave case by setting either *n_y_* or *n_z_* to 0.

#### 3.1.1. Particle Concentration

**Figure 2:**
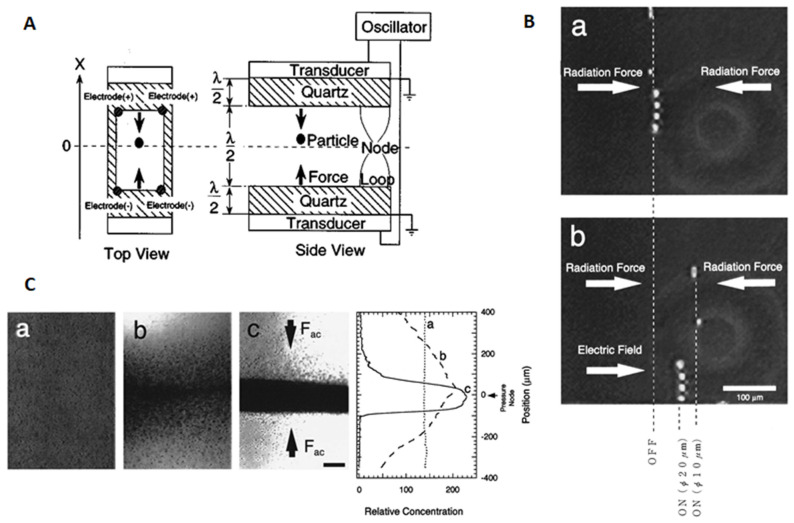
Acoustic microparticle concentration in rectangular cross-section glass capillaries *w* = 2.78 mm). (**A**) Schematic diagrams of the rectangular glass capillary-based ultrasonic cell containing electrodes. (**B)** A comparison of the steady-state distribution of 10 and 20 μm polystyrene particles under the effect of acoustic radiation force alone (**a**) and both acoustic radiation and electrostatic forces (**b**). Reprinted with permission from [40]. (**C**) In a similar ultrasonic cell to **A**, erythrocytes were concentrated to acoustic pressure node at the center of the fluid channel. Reprinted with permission from [41].

As one of the most used channels, rectangular cross-section glass capillaries were firstly used for ultrasonic concentration of microparticles. As early as 1995, Yasuda et al. [40] designed an ultrasonic cell containing electrodes to concentrate and separate microparticles of different sizes, as shown in Figure 2A. They attached two PZTs to the opposite side walls of the rectangular cross-section glass capillary (*w* = 2.78 mm) and excited the transducers through a signal generator. Electric signals were converted into mechanical vibrations, which are translated into ultrasonic waves propagating in the channel. Two ultrasonic waves with the same amplitude and frequency but opposite directions interfered in the channel to generate standing waves. Particles of diameters of 10 and 20 μm were focused to the single acoustic pressure node by acoustic radiation forces and were then separated after a uniform electric field was applied, as shown in Figure 2B. Later on, based on the above ultrasonic cell, Yasuda et al. [41] achieved concentration of erythrocytes (shown in Figure 2C) by a 500 kHz ultrasonic standing wave. They also demonstrated that acoustic radiation force is effective for concentrating living cells without notable damage under a cavitation-free condition.

#### 3.1.2. Particle Alignment and Patterning

**Figure 3:**
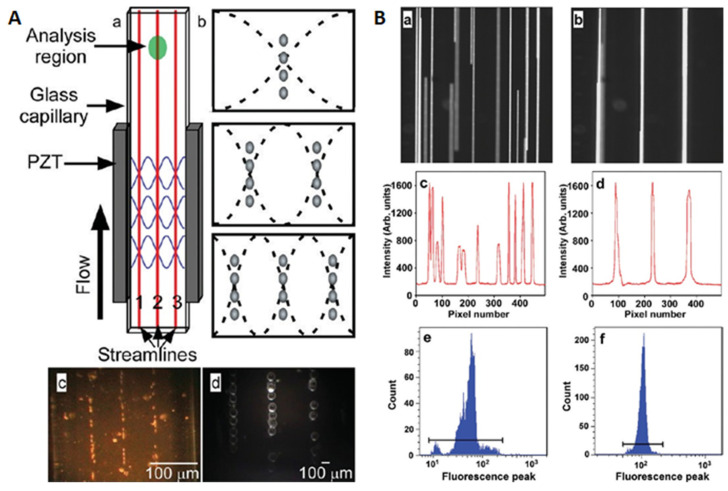
Multinode ultrasonic microparticle alignment in a rectangular cross-section glass capillary. (**A**) shows a schematic drawing of the device (a), the principle of particle alignment (b), and the alignments of 10 μm particles in a *w* × *h* = 1 × 0.1 mm capillary (c) and 107 μm polystyrene particles in a 2 × 0.2 mm capillary (d). (**B**) analyses of multinode acoustic particle alignment in a 1 × 0.1 mm glass capillary were presented. Reprinted with permission from [42].

Rectangular cross-section glass capillaries were also used for ultrasonic alignment and patterning of particles. Arranging cells into desired patterns is especially important for biomedical research (e.g., cell culture). Similar to the principle of particle concentration, ultrasonic standing waves are generated for the alignment and patterning of particles, where the ultrasonic frequencies can be adjusted to customize the number of pressure nodes in the fluid channel. Piyasena et al. [42] developed a multinode acoustic particle alignment device using rectangular cross-section glass capillaries. As shown in the upper half of Figure 3A, two PZTs were glued to the short sidewalls of the rectangular glass capillary to form multinode standing waves in the fluid channel. When the transducers were actuated, microparticles of different sizes (e.g., 10 and 107 μm in diameter) were aligned to parallel acoustic pressure nodes generated in the channels of two rectangular glass capillaries. As shown in Figure 3B, by precision alignment/focusing of microspheres, they also demonstrated the potential of such ultrasonic flow cells for the development of high throughput, parallel flow cytometers.

#### 3.1.3. Particle Trapping and Transportation

**Figure 4:**
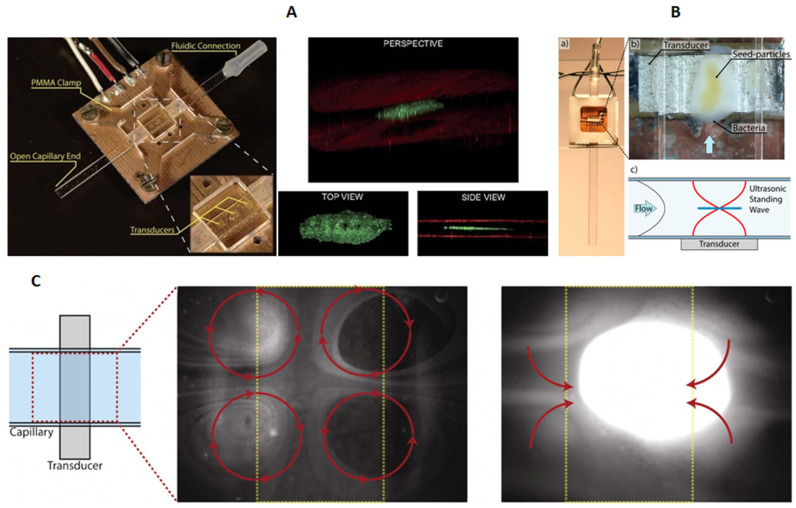
Acoustic trapping of micron and submicron particles in rectangular cross-section glass capillaries. (**A**) Three-dimensional acoustic trapping of microparticles in a rectangular glass capillary (*w* × *h* = 2 × 0.1 mm). Reprinted with permission from [44]. (**B**) Seed-particle enabled acoustic trapping of bacterial in a rectangular glass capillary (2 × 0.2 mm). Reprinted with permission from [46]. (**C**) Acoustic trapping of nanoparticles in a rectangular glass capillary (2 × 0.2 mm) by a four-quadrant acoustic streaming pattern at low and high concentrations. Reprinted with permission from [45].

Furthermore, acoustic trapping and transportation of particles have been reported in planar rectangular cross-section glass capillaries. Acoustic trapping is a useful method for handling biological samples in microfluidic systems and has been proved to be non-invasive to the cells exposed in ultrasonic waves [43]. Hammarström et al. [44] showed non-contact acoustic cell trapping in a planar glass capillary (*w* × *h* = 2 × 0.1 mm). As shown in Figure 4A, 3D aggregation of 4.2 μm fluorescein isothiocyanate tagged polystyrene beads were formed above the active transducer zone by a localized standing wave field. Later on, Hammarström et al. [45,46] developed a similar device, composed of a planar glass capillary (*w* × *h* = 2 × 0.2 mm) and a PZT, for acoustic trapping of nanoparticles and bacteria (*E. coli*). They found that, without using seed particles, particle concentration plays an important role in acoustic trapping of submicron particles [45]. At high particle concentrations, continuous enrichment of 490 nm polystyrene particles was achieved by localized acoustic streaming vortices (i.e., the four-quadrant transducer plane streaming [32]), shown in Figure 4B. With seed particles, continuous trapping of submicron particles and bacteria at significantly lower concentrations were accomplished (see Figure 4C), which provided correct identification in 12 out 12 cases of *E. coli* positive blood cultures [46].

In a similar device, which consists of a planar rectangular glass capillary (*w* × *h* = 2 × 0.2 mm) and a PZT, Fornell et al. [47] showed acoustic trapping of cell-laden hydrogel droplets. They reported that the droplet cluster can be retained at flow rates of up to 76 µL/min. Recently, Fornell et al. [48] further studied the physics behind acoustic trapping in the rectangular glass capillary (*w* × *h* = 2 × 0.2 mm) and found that binary acoustic trapping can be achieved by increasing the density of the fluid in the trapping channel when the density of particles is higher than the fluid, which enabled selective trapping of melamine particles from a mixture of melamine particles and polystyrene particles in a high-density fluid.

**Figure 5:**
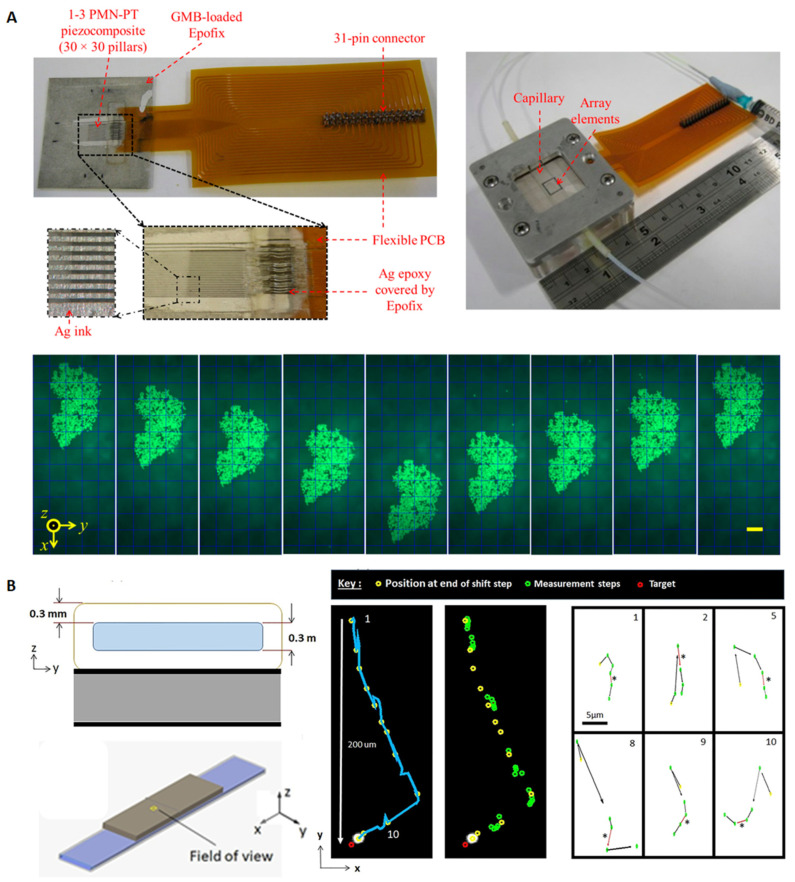
Acoustic trapping and transportation of microparticles in rectangular cross-section glass capillaries (*w* × *h* = 6 × 0.3 mm). (**A**) Acoustic trapping and transportation of microparticles with a PZT array-controlled glass capillary. Microparticles suspended in fluid were trapped at the center of the channel toward the acoustic velocity maximum centered above the set of active PZT elements and transported along the channel by switching the active elements. Reprinted with permission from [51]. (**B**) Acoustic transportation of single microparticles, where the right hand side shows an exemplified steering manipulation of a levitated 10 µm bead over a 200 µm distance from top to bottom towards the target position using the direction method. Reprinted with permission from [52].

Ultrasonic transportations of microparticle clusters have been achieved in planar rectangular glass capillaries using transducer arrays. Glynne-Jones et al. [49] developed a 12-element 1D transducer array coupled to a rectangular cross-section glass capillary (*w* × *h* = 6 × 0.3 mm). Microparticles suspended in fluid were firstly trapped at the center of the channel toward the acoustic velocity maximum centered above the set of active PZT elements, and then transported along the channel by switching the active elements. Later on, Qiu et al. [50,51] designed a similar particle transportation device composed of a same size of rectangular glass capillary and a 30-element ultrasonic array, shown in Figure 5A. The lower part of Figure 5A demonstrated 3D trapping of microparticles and transportation of microparticles in the *y*-direction of the fluid channel. As can be seen, an agglomerate of microparticles was formed by activating adjacent ultrasonic elements of the array and was transported along the channel by altering the activated elements. They also demonstrated 2D patterning of microparticle clusters using a 2D matrix ultrasonic array.

Ultrasonic transportation of single microparticles has also been demonstrated in a planar rectangular glass capillary (*w* × *h* = 6 × 0.3 mm) by Shaglwf et al. [52], who combined the camera feedback and PC algorithm to switch the frequency of the transducer to achieve closed loop control. Two different methods, i.e., the combined forces method and the direct method, with trade-offs for more accurate paths vs. enhanced speed, respectively, were explored for single particle steering. An exemplified steering manipulation of a levitated 10 μm bead over a 200 μm distance from top to bottom towards the target position using the direction method is shown in Figure 5B.

### 3.2. Square Cross-Section Channel Glass Capillary

The fundamental theory of acoustic particle manipulation in square cross-section channels is similar to that in rectangular channels described in the previous section. For acoustic resonances established in cross-section of the channel, the resonant frequency follows

(18) $$f_{y,z}=\frac{c_{0}}{2a}\sqrt{n_{y}^{2}+n_{z}^{2}}.$$

#### 3.2.1. Particle Alignment and Patterning

**Figure 6:**
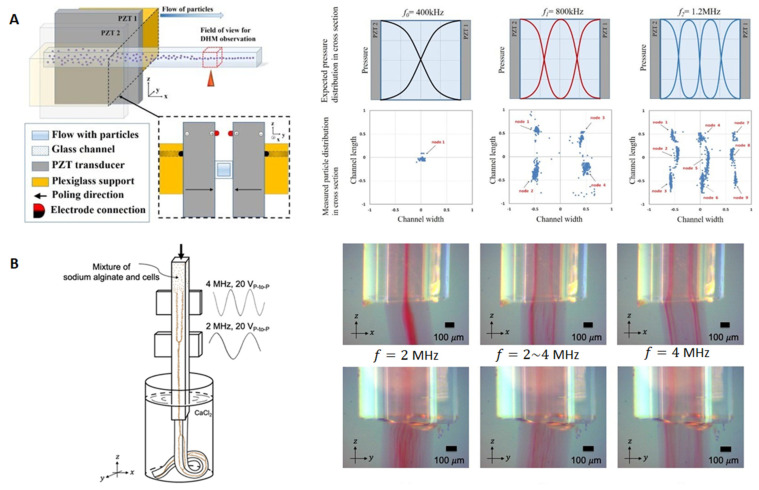
Acoustic alignment and patterning of microparticles in square cross-section glass capillaries using BAWs. (**A**) Particle alignment and patterning in a square glass capillary (*a* = 2 mm) by acoustic waves generated from two opposing PZTs. Reprinted with permission from [53]. (**B**) Acoustic particle and cell patterning in hydrogel in a square glass capillary (*a* = 0.4 mm) at cavity modes generated from a single PZT. Reprinted with permission from [54].

Square cross-section glass capillaries have been used for acoustic concentration and patterning of micro- or nanoparticles in literature. Perfetti and Iorio [53] reported one of the early studies of acoustic particle manipulation in square glass capillaries. As shown schematically in the left hand side of Figure 6A, they developed a device consisting of a square glass capillary (a=2 mm) and two PZTs which were glued to the opposite lateral walls of the capillary. They recorded the particle positions by a digital holographic microscope and imported their 3D coordinates into MATLAB for statistical analysis. 

As seen from Figure 6A (right), as the driving frequency increased, various patterns of microparticles were formed in cross-section of the fluid channel. Based on the same principle, Li et al. [55] recently conducted a similar acoustic particle alignment experiment in a square glass capillary (a=0.25 mm). They demonstrated 2D concentration of microparticles by actuation of two vertically placed PZTs, and reasonable focusing performances were reported with flow rates up to 100 µL/min. Similarly, Gonzalez et al. [56] showed acoustic enrichment of blood cells from whole blood in a square glass capillary (*a* = 0.7 mm) by a half-wavelength standing wave generated in the lateral direction of the channel. Recently, Koo et al. [54] showed acoustic cell patterning in hydrogel in a square glass capillary (*a* = 0.4 mm), shown in Figure 6B. Different to ultrasonic excitations in the previous work, they generated 2D cavity modes by a single PZT. As a result, single or quadruple streams of microparticles were generated at actuation of 2 and 4 MHz, respectively. Additionally, apart from the standing wave technique described above, Jonas et al. [57] demonstrated that it was possible to push microparticles to the side wall of a square cross-section glass capillary (*a* = 0.08 mm) when the characterized size of the channel (i.e., *a*) is smaller than a quarter wavelength of the acoustic wave generated in the fluid channel. The combining effects of the shearing force induced by acoustic streaming and the acoustic radiation force that pushes the cells to the channel wall could lead to sonoporation of cells. They reported delivery of plasmid DNA to immortalized and primary human cell types with a throughput of 200,000 cells/min.

#### 3.2.2. Particle Focusing

**Figure 7:**
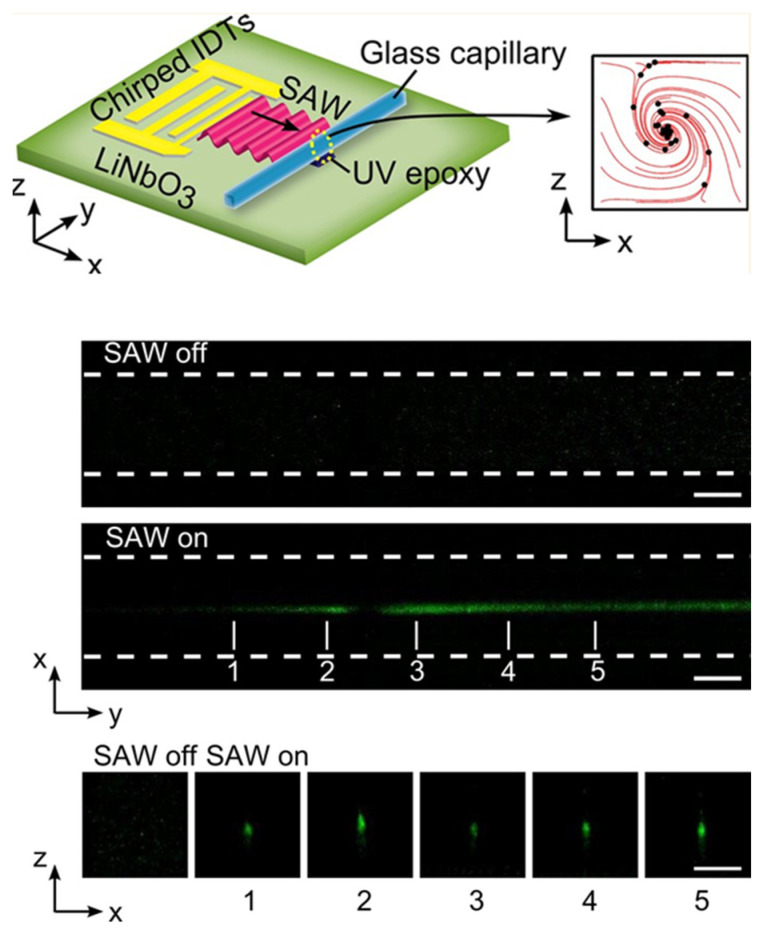
Acoustic focusing of nanoparticles in square cross-section glass capillaries by SAWs. Chirped interdigital transducers (IDTs) generate SAWs, which propagate into the capillary and generate acoustic streaming with a single vortex. 2D focusing of nanoparticles down to 80 nm was reported. Reprinted with permission from [58].

Mao et al. [58] reported 2D enrichment of nanoparticles in a square cross-section glass capillary (*a* = 0.2 mm) using SAW-induced acoustic streaming, as shown in Figure 7. Different from the configuration of normal BAW particle manipulation devices described above, the device in Mao et al.’s work mainly consists of a lithium niobate (LiNbO_3_) substrate with chirped IDTs and a square glass capillary. When the transducers were excited, SAWs are generated, propagate into the capillary and generate acoustic streaming with a single vortex. As a result, 2D focusing of submicron particles to the center of the channel (see Figure 7) were realized under the combining effects of acoustic streaming and acoustic radiation forces. Using this method, they achieved acoustic focusing of 500–100 nm diameter polystyrene particles, as well as 200 and 80 nm silica particles. Here, for 2D nanoparticle focusing, acoustic streaming plays a major role as the acoustic radiation force is much weaker than the streaming-induced drag force and it could not be achieved with the acoustic radiation force alone.

#### 3.2.3. Particle Trapping for Deformability Analyses

**Figure 8:**
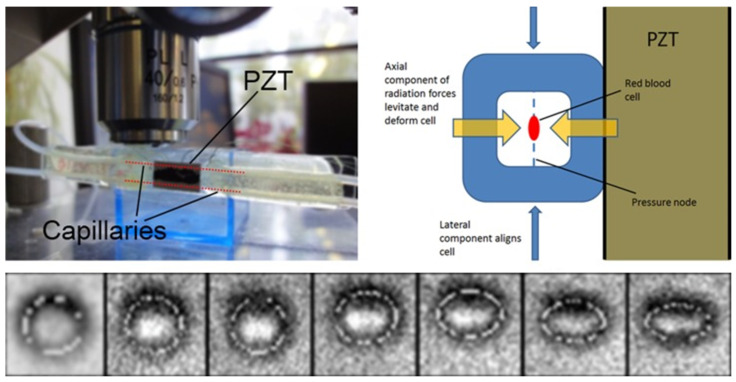
Acoustic trapping of single cells in a square cross-section glass capillary (*a* = 0.1 mm). Single red blood cells were trapped at the center of the fluid channel by a half-wavelength standing wave field. With the increase of acoustic pressure amplitude, deformation of a red blood cell was increased towards an ellipsoid. Reprinted with permission from [59].

In a square cross-section glass capillary (*a* = 0.1 mm), Mishra et al. [59] demonstrated deformation of single red blood cells using acoustic radiation forces. As shown in the upper half of Figure 8, a single PZT was used to create a half-wavelength standing wave field in cross-section of the fluid channel, in which cells were trapped to the channel center and deformations of single red blood cells were observed. They showed with numerical modeling that, at the acoustic pressure nodal plane where single cells were levitated, acoustic radiation forces exert a net outward stress at all points over the cell membrane, which accounted for the deformation of cells. Increasing pressure amplitude (i.e., increasing acoustic radiation force) results in increasing amounts of cell deformation (see lower half of Figure 8) and deformations up to an aspect ratio of 1.35, which is comparable to optical tweezer-induced deformations, were reported.

### 3.3. Circular Cross-Section Channel Glass Capillary

As mentioned in the introduction section of this review, a large number of materials have been used for building ultrasonic resonators. However, to date, most of the acoustic particle manipulations in circular cross-section fluid channels were accomplished in glass capillaries. The reason is that glass is the rare occasion that it is easy to form cylindrical channels and to generate strong lateral acoustic resonances, compared to other materials such as silicon, metal and polymers. The acoustic resonances generated in the cross-section of cylindrical channels are usually labeled (m, *n*) modes, where *m* and *n* represent the number of nodal diameters and circles, respectively. The resonant frequency of a (*m*, *n*) mode is described by

(19) $$f_{mn}=k_{mn}\frac{c_{0}}{\pi d},$$

where $k_{mn}$ is *n*th zero of Bessel function of the first kind of order *m*, $J_{m}\left(\cdot \right)$. For a 1D standing wave generated in the x direction of the fluid channel, the theoretical resonant frequency follows the one described for rectangular channels in Equation (17).

#### 3.3.1. Particle Focusing

**Figure 9:**
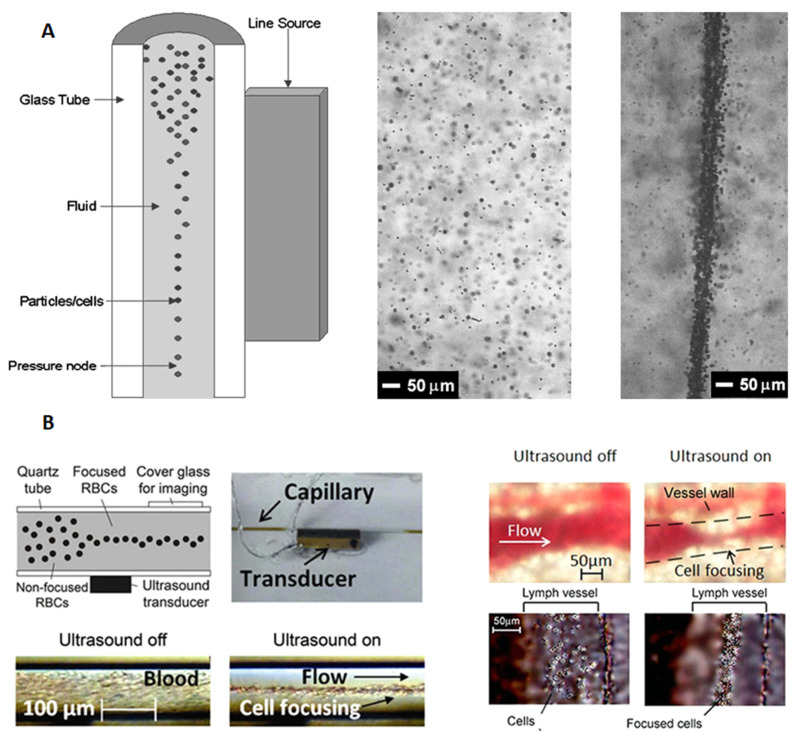
Acoustic focusing of microparticles in circular cross-section glass capillaries. (**A**) Ultrasonic microparticle concentration in a soft glass capillary (*d* = 2.2 mm). Reprinted with permission from [61]. (**B**) In vitro and in vivo ultrasonic focusing of cells. In vitro (**left**): acoustic focusing of blood cells was performed in a circular quartz capillary (*d* = 0.1 mm). In vivo (**right**): acoustic cell focusing in blood in a mouse ear vessel and acoustic focusing of cells (WBCs) in a 180 μm diameter mouse mesenteric lymph vessel. Reprinted with permission from [65].

Circular cross-section glass capillaries have been widely used for acoustic concentration/focusing of microparticles. Typically, in such a device, a (1, 0) mode is generated in cross-section of the fluid channel where the primary and lateral acoustic radiation forces tend to move microparticles into the center of the channel [60]. An early relevant work was performed by Goddard and Kaduchak [61], who designed an acoustic microparticle focusing device that is composed of a circular cross-section glass capillary and a PZT, shown in Figure 9A. They conducted microparticle focusing experiments in two glass capillaries, i.e., a soft glass tube (d=2.2 mm) and a quartz tube (*d* = 2 mm), and good focusing performances were obtained for both cases. Moreover, they conducted further experiments to test the application of ultrasonic particle focusing on flow cytometer and demonstrated that acoustic focusing can dramatically assist the hydrodynamic focus and improve the efficiency of flow cytometer [62,63,64]. Galanzha et al. [65] performed in vitro acoustic cell focusing in a circular cross-section glass capillary (d=0.1 mm) and in vivo acoustic focusing of circulating cells in living animals. As shown in Figure 9B, ultrasonic cell focusing in blood and lymph flow in the ear and mesentery of mice were achieved.

**Figure 10:**
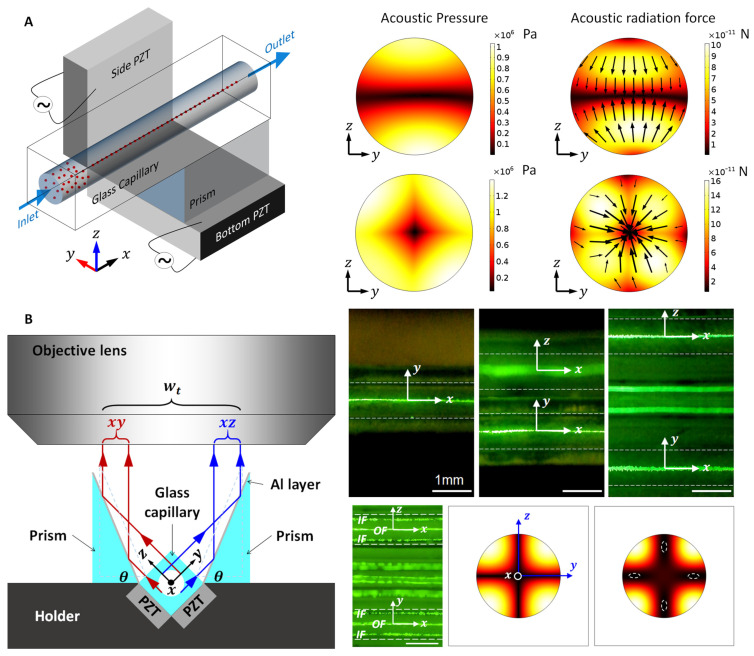
(**A**) Two-dimensional acoustofluidic concentration of microparticles in a glass capillary whose cross-section is square outside and circular inside *d* = 0.9 mm). Microparticles were rapidly focused to the center of the channel in both *y*- and *z*-directions by two orthogonal (1, 0) standing wave modes. Reprinted with permission from [66]. (**B**) An optical imaging system enabled by two 67.5° right triangular prisms for the measurements of three-dimensional microparticle acoustophoresis. As shown, this method is more efficient than conventional microscopy, direct measuring without prism or with an isosceles right triangular prism. The lower right shows the imaging of two-dimensional microparticle patterning, which can be used to identify the acoustic resonances generated in the cross-section of the fluid channel. Reprinted with permission from [67].

Recently, Lei et al. [66] reported a 2D ultrasonic particle focusing device consisting of two orthogonally placed PZTs and a circular cross-section glass capillary (*d* = 0.9 mm), shown schematically in Figure 10A. To make the most of orthogonally ultrasonic excitations, a circular glass capillary with a square outer cross-section was designed. It was shown that a nearly uniform distribution of acoustic radiation force field was obtained when two orthogonal (1, 0) modes were generated in cross-section of the channel and the efficiency of particle concentration was much improved when compared to the excitation of a single PZT. It was demonstrated that 2D focusing of 10 μm polystyrene particles was achieved for flow rate up to 400 µL/min. Later, Lei et al. [67] designed a dual 67.5° prisms method to characterize 3D microparticle acoustophoresis in circular cross-section glass capillaries. The working mechanism is presented in Figure 10B, which shows that microparticle acoustophoresis on both horizontal and vertical planes of the fluid channel can be simultaneously observed and characterized, making it more powerful than conventional microscopy, e.g., direct measurement from an upright or inverted microscope or measuring with a right triangular prism, especially on the characterization of higher modes generated in the channel (see lower right of Figure 10B).

#### 3.3.2. Particle Patterning

**Figure 11:**
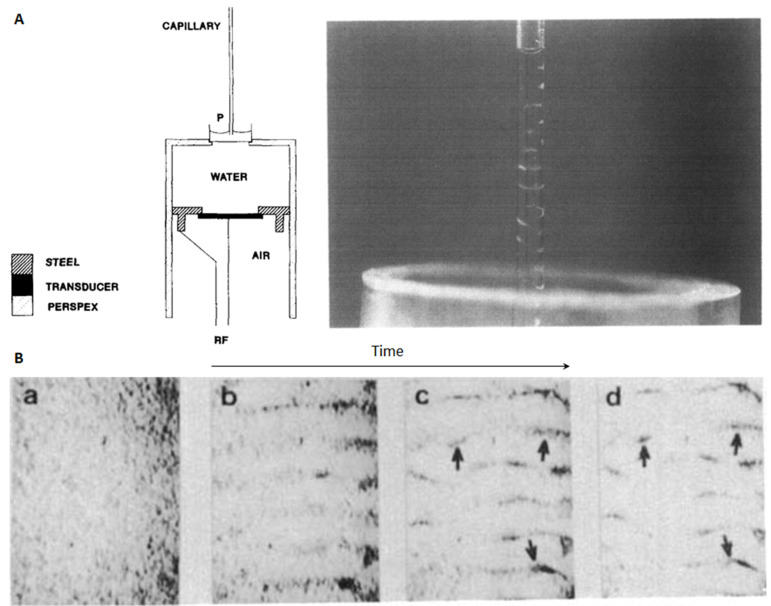
Acoustic microparticle patterning in the flow direction of circular cross-section glass capillaries. (**A**) Acoustic agglutination of *Legionella pneumophila* in a circular glass capillary (*d* = 1 mm) containing antiserum. Presented is a photograph of the agglutinates 20 s after exposure to ultrasound. Reprinted with permission from [68]. (**B**) In a device similar to **A**, erythrocytes were well patterned by ultrasound along the fluid channel of the glass capillary (*d* = 13 mm). Reprinted with permission from [69].

Before the concept of acoustic tweezers was proposed [11], ultrasonic patterning of particles in circular cross-section glass capillaries had been studied. In 1989, Jepras et al. [68] and Coakley et al. [69] reported acoustic particle patterning in a similar device (see Figure 11A). As shown, the transducer was fixed by a metal frame and there was a water area between the transducer and a circular cross-section glass capillary. In Jepras et al.’s work, one end of the glass capillary (of three different sizes, i.e., *d* = 1, 2, 5 mm) was immersed in a water tank and the other end was sealed with plasticine; while a coverslip was placed on top of the capillary (d=13 mm) acting as a sound-reflecting surface in Coakley et al.’s work. The acoustic waves propagate through the water into the tank and form a standing wave field in the capillary along the fluid channel, and thus particles or erythrocytes were patterned to acoustic pressure nodes in the vertical direction of the channel, as shown in Figure 11A,B.

Another acoustic particle patterning device, consisting of a ring-shaped transducer and a circular cross-section glass capillary (d=2 mm), has been reported and widely used in the agglutination of particles for biomedical research [70,71,72,73,74,75,76]. In this device, a cylindrical glass capillary was placed in the center of the ring-shaped transducer, leaving a water gap between them. It was found that the particles or droplets suspended in the fluid could be driven to the pressure nodes forming various patterns in the capillary. The patterns that the particles formed in cross-section of the channel, however, to some extent were randomly presented and were not well backed by theoretical or numerical solutions.

**Figure 12:**
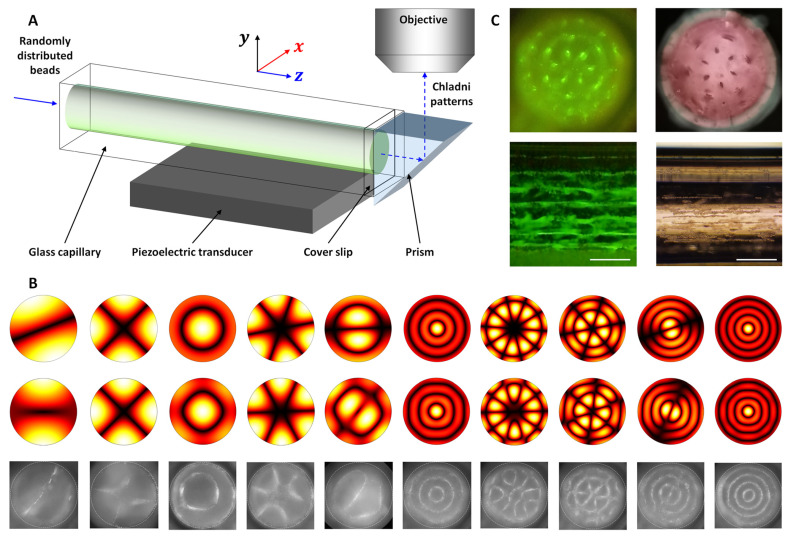
Acoustic microparticle patterning in cross-section of a circular glass capillary (*d* = 1.6 mm). (**A**) Schematic of the acoustofluidic device and imaging system. (**B**) The predicted acoustic resonances and the measured patterns of 10 µm particles in cross-section of the channel. (**C**) Further trapping of 10 μm polystyrene particles (**left**) and Hela cells (**right**). Reprinted with permission from [77].

Recently, Lei et al. [77] presented theoretical and experimental studies on ultrasonic patterning of microparticles in cross-section of a cylindrical channel. Figure 12A shows schematically the device and the imaging system, mainly composed of a circular (*d* = 1.6 mm) cross-section glass capillary (with a square outer cross-section) and a PZT. The capillary was sealed at one end with a cover slip where an isosceles right triangular prism was attached for the observation and characterization of microparticle acoustophoresis in cross-section of the fluid channel. As shown in Figure 12B, the observed microparticle patterns compare well with those predicted from numerical simulations. These patterns were referred to as nonconventional Chladni patterns since they are very close to the Chladni figures [78] formed over a vibrating circular plate [79]. They also demonstrated trapping of microparticles and Hela cells to Gorkov potential minima with further ultrasonic excitations, shown in Figure 12C.

#### 3.3.3. Particle Trapping

**Figure 13:**
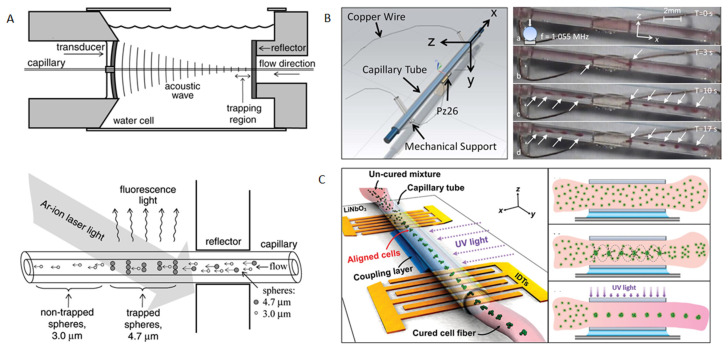
Acoustic trapping of microparticles in circular cross-section glass capillaries. (**A**) Trapping in a circular glass capillary *d* = 0.075 mm) using a focusing transducer. The 4.7 µm particles were trapped along the channel from a mixture of 4.7 and 3 µm particles. Reprinted with permission from [80]. (**B**) Trapping in a circular glass capillary *d* = 0.085 mm) using a single PZT. The times indicate how long the microparticles were trapped into clusters along the fluid channel. Reprinted with permission from [82]. (**C**) Trapping in a circular glass capillary (*d* = 0.1 mm) using SAWs. Beads and Hela cells were trapped within a viscous polymer solution, which could then be polymerized and extracted within a polyethylene tube. Reprinted with permission from [83].

Acoustic trapping of microparticles in circular cross-section glass capillaries have been achieved via different means of ultrasonic excitation. Wiklund et al. [80] designed an ultrasonic particle trapping device that mainly consists of a circular glass capillary (*d* = 75 μm) and an 8.5 MHz hemispherical focusing transducer, shown in Figure 13A. A standing-wave pattern was created inside the capillary from the focusing transducer by reflection from a plane acoustic reflector. To minimize acoustic reflection losses, the whole device was immersed in water. Inside the capillary, acoustic radiation forces and viscous drag forces act in opposite directions on the spheres, resulting in size-selective trapping and in-flow separation. As shown schematically in Figure 13A, 4.7 µm particles were trapped along the channel from a mixture of 4.7 and 3 µm particles. On the basis of a similar device, they further enhanced the ability to detect ultra-low concentrations of proteins by combining ultrasonic trapping and capillary electrophoresis [81]. Gralinski et al. [82] showed acoustic trapping of microparticles in a device composed of a circular glass capillary and a single PZT, shown in Figure 13B. They demonstrated that, in absence of flow, the suspending particles in the capillary were firstly concentrated to the channel center and then trapped into clumps along the fluid channel. In presence of flow, it was demonstrated that the particles could be held in a flow of up to 0.833 µL/s. Lata et al. [83] reported an acoustic particle trapping method based on SAWs, as shown in Figure 13C. The trapping device consists of a lithium niobate substrate, chirped IDTs and a circular glass capillary (*d* = 0.1 mm). The capillary was glued to the substrate and placed parallel to the propagation of SAWs. They demonstrated that in their device cells could be trapped and patterned within a viscous polymer medium to create cellular fibers which can be used to build complex 3D tissue architectures.

#### 3.3.4. Particle Separation

In a mixture of various cells, the separation of the same type of cells has become an important issue in biomedical research and many reviews about it have been published recently [84,85,86,87,88,89]. It is a prerequisite technique for studying the properties of the same type of cells in a mixed medium. Thereinto, separation of various particles by acoustic waves mostly relies on the differences of axial acoustic radiation forces exerted on the particles [90]. In circular cross-section glass capillaries, acoustic separation of microparticles of different particle sizes or compressibility have been demonstrated. Araz et al. [91] designed an acoustic particle separation device consisting of a circular glass capillary and a transducer with bond grooves and cantilevers. They demonstrated acoustic collection/trapping of microparticles to pressure nodes and separation of microparticles of different properties. The following particle separating experiments were performed in their device: (1) three micrometer polystyrene beads and air bubbles mixture were separated as beads and air bubbles were collected at the pressure nodes and antinodes, respectively; (2) 3 and 10 micron fluorescent microbeads were separated by frequency hopping; (3) white and red blood cells were separated from blood plasma and collected at pressure nodes. One of the main drawbacks of their microparticle separation system is that the particles were separated in a static flow condition such that it would be difficult to collect the different particles that have been separated in the capillary.

## 4. Discussion and Conclusions

We summarized in this paper the recent advancement of ultrasonic particle manipulation (UPM) in various cross-section glass capillaries. The established and emerging glass capillary-based UPM devices, the corresponding applications and the underlying mechanisms were briefly introduced. A summary of the use of various cross-section glass capillaries for UPM is presented in Table 1. These different types of glass capillaries are easy to fabricate but the choice of glass capillary for UPM should be dependent on the potential applications. In general, rectangular channels are more suitable for 1D alignment and patterning of microparticles, circular channels more easily achieve fast 2D focusing and 3D trapping of microparticles, and square channels are potentially better for 2D alignment or patterning of microparticles, as well as acoustic streaming-based submicron particle focusing.

To conclude, merits of glass capillary for UPM include but are not limited to: (1) whole fluid channel—it requires no additional bonding of different components, which is an issue that has to be concerned in other types of microfluidic channels; (2) high acoustic quality factor and high acoustic impedance difference to water—it allows for the building of strong 1D, 2D or 3D acoustic resonances [30]. (It is noteworthy that to date UPM has been demonstrated in glass capillaries that are made out of several types of glass materials, such as quartz, borosilicate and soda lime glasses. The properties (e.g., compressional and transversal sound speeds and density) of different glass materials may vary slightly, but do not have a significant effect on the performance of UPM); (3) optically transparent—it theoretically enables observation and characterization of microparticle acoustophoresis in the channel from any direction. Different microscopic imaging methods could be designed according to the structure of the glass capillary and the specific applications, which could sometimes avoid using the expensive and slow confocal microscopy [92] and even provide better characteristics of acoustophoresis [67,77]; (4) cost effective—off-the-shelf disposable glass capillary provides a significant device simplification, making cleanroom microfabrication obsolete.

Although a large number of glass capillary-transducer devices have been constructed for various kinds of particle manipulation, efforts shall be made to make further progress of fundamental acoustofluidic research and to promote this type of UPM devices for real applications in e.g., biomedical and biochemical research. For glass capillary-based BAW tweezers, more versatile and efficient UPMs remain to be explored. One suggestion is to optimize the acoustofluidic fields in the channel for particle manipulation through careful capillary shape control, which would be possible to implement as it is easy to shape the inner or outer cross-section of glass capillary. Then, as above demonstrated, only a few studies in the literature, e.g., those from the Tony Huang group [58,83], have shown UPM in glass capillaries using SAWs. Most previous SAW-based tweezers manipulate particles in the horizontal (i.e., *xy*) plane of PDMS channels, glass capillaries could additionally provide sound reflections and form standing waves in the vertical direction of channel, i.e., z, which could enable 3D manipulation of microparticles. We expect that more work could supplement UPM from a combination of SAWs and glass capillaries. In addition, very few studies in the literature showed particle manipulation in glass capillaries by integrating acoustic radiation force with other force fields (e.g., magnetic, electric, hydrodynamic and optical forces). We would expect to see clever glass capillary-based designs that can combine acoustic particle manipulation with other techniques or force fields, which may enable operations that would not be easily performed by either force field alone [93].

## Figures and Tables

**Table 1:** Summary of the use of glass capillaries for ultrasonic particle manipulation.

Types (CS)	Dimensions (mm)	Manipulation	Particles	Mechanism
Rectangular	*w =* 2.78	Concentration	PS (10, 20 μm)	SBAW: a single PZT [40]
	*h* × *w*: 1 × 0.1, 2 × 0.2	Alignment	PS (10, 107 μm)	SBAW: a pair of PZTs [42]
	*h* × *w*: 2 × 0.1, 2 × 0.2	Trapping	PS (4.2, 10 μm), RBC	SBAW: a single PZT [44,47]
			PS (0.49, 0.11 μm), bacteria	Acoustic streaming [45,46]
	*h* × *w*: 6 × 0.3	Transportation	PS (10 μm)	SBAW: arrayed PZTs [49,50,51]
				SBAW: a single PZT and steering algorithm [52]
Square	*a*: 2, 0.7, 0.4	Patterning	PS (3 μm), blood cells	SBAW: a pair of PZTs [53,55,56]
			PS (10 μm), fibroblasts	SBAW: a single PZT [54]
	*a*: 0.2	Focusing	PS (0.1–0.5 μm), silica (0.2 and 0.08 μm)	SSAW: a single IDT [58]
	*a*: 0.1	Trapping for deformability analyses	RBC	SBAW: a single PZT [59]
Circular	*d*: 0.1, 2, 2.2,	Focusing	PS (6.8, 10, 25 μm), RBC, WBC	SBAW: a single PZT [61,65]
	*d*: 1, 13	Patterning	*Legionella pneumophila*, RBC, bacteria	SBAW: a single PZT [68,69]
	*d*: 0.075, 0.1, 0.85	Trapping	Latex (3, 4.7 μm), PS (5 μm)	SBAW: a single PZT [80,82]
			HeLa S3, MC3T3-E1, PC12 Adh cells, PS (10 μm)	SSAW: a pair of IDTs [83]
	*d*: 0.1	Separation	PS (0.3, 3, 10 μm), air bubble, bacteria, WBC, RBC	SBAW: a single PZT [91]
Square-circular	*d*: 0.9	Focusing	PS (10 μm)	SBAW: two orthogonal PZTs [66]
	*d*: 1.6	Patterning, trapping	PS (10 μm), Hela cells	SBAW: a single PZT [77]

Abbreviations: CS, cross-section; SBAW, standing bulk acoustic wave; SSAW, standing surface acoustic wave; RBC, red blood cell; WBC, white blood cell; PS, polystyrene; PZT, piezoelectric transducer; IDT, interdigital transducer.
